# Open-Label, Randomized Study to Assess Safety and Efficacy of Slow and Accelerated Switching to Xanomeline/Trospium from Standard of Care Atypical Antipsychotics in Participants with Schizophrenia

**DOI:** 10.1192/bjo.2026.11092

**Published:** 2026-06-30

**Authors:** David Walling, Naomi Marbot, Lara Shirikjian, Edwin Gomez, Lauren White

**Affiliations:** 1 CenExel, Los Alamitos, USA; 2 Bristol Myers Squibb, Princeton, USA

## Abstract

**Aims:**

Antipsychotics (APs) may cause significant adverse events (AEs) resulting in treatment discontinuation and relapse. Xanomeline/trospium chloride (KarXT), a dual M1/M4 muscarinic receptor agonist combined with a peripheral pan muscarinic receptor antagonist, has a favourable side-effect profile, potentially lowering the risk of cardiometabolic and extrapyramidal AEs. Healthcare providers may benefit from clinical data and guidance on switching patients from traditional APs to KarXT.

**Methods:**

This 8-week, multicenter, randomized, open-label, outpatient trial assessed efficacy and safety of a switch from atypical APs to KarXT monotherapy in adults with schizophrenia based on criteria from the Diagnostic and Statistical Manual of Mental Disorders, Fifth Edition. Participants were randomized 1:1 to 2 treatment groups: slower transition from AP to KarXT over 4 weeks (25% reduction in AP dose/week) or faster transition from AP to KarXT over 2 weeks (50% reduction in AP dose/week). Participants in both treatment groups followed the same titration schedule, initiating KarXT at 50 mg/20 mg twice daily for 1 week and uptitrating to a target maintenance dosage of 125 mg/30 mg over 8 weeks. The primary endpoint was all-cause discontinuation of KarXT. Secondary endpoints included change from baseline to week 8 in Positive and Negative Syndrome Scale (PANSS), Clinical Global Impressions-Severity (CGI-S), and Personal and Social Performance (PSP) scores. Safety endpoints included KarXT AE-related discontinuations and AE incidence.

**Results:**

Fifty-three participants were enrolled in the slower transition group and 52 in the faster transition group; 86% of participants completed 8 weeks of treatment, with discontinuation rates of 15.1% (n=8) and 13.5% (n=7) in the slower and faster transition groups, respectively. No participant discontinued due to lack of efficacy. Mean changes in PANSS total scores from baseline to week 8 were −4.2 (slower transition group) and −3.1 (faster transition group). Mean change in CGI-S score was −0.2 in both transition groups. From baseline to week 8, mean PSP scores increased by 1.1 and 0.7 in the slower and faster transition groups, respectively. Forty-nine per cent of participants had ≥1 treatment-emergent AE (TEAE); none were serious. In the slower and faster transition groups, 1 (1.9%) and 2 (3.8%) participants, respectively, discontinued treatment early due to TEAEs.

**Conclusion:**

Both slower and faster transitions from stable AP treatment to KarXT were generally safe and effective, suggesting that either transition method may be considered. These results aid clinical decision-making, providing healthcare providers with evidence-based guidance on how to switch to KarXT from oral atypical APs.

